# Alcohol Acutely Antagonizes Refeeding-Induced Alterations in the Rag GTPase-Ragulator Complex in Skeletal Muscle

**DOI:** 10.3390/nu13041236

**Published:** 2021-04-09

**Authors:** Lacee J. Laufenberg, Kristen T. Crowell, Charles H. Lang

**Affiliations:** 1 Department of Surgery, Penn State College of Medicine, Hershey, PA 17033, USA; ellaufenberg@pennstatehealth.psu.edu (L.J.L.); kcrowell@bidmc.harvard.edu (K.T.C.); 2 Beth Israel Deaconess Medical Center, Department of Surgery, Boston, MA 02215, USA; 3 Department of Cellular & Molecular Physiology, Penn State College of Medicine, Hershey, PA 17033, USA

**Keywords:** protein synthesis, leucine: LAMPTOR1: V-ATPase, mTORC1, anabolic resistance

## Abstract

The Ragulator protein complex is critical for directing the Rag GTPase proteins and mTORC1 to the lysosome membrane mediating amino acid-stimulated protein synthesis. As there is a lack of evidence on alcohol’s effect on the Rag-Ragulator complex as a possible mechanism for the development of alcoholic skeletal muscle wasting, the aim of our study was to examine alterations in various protein–protein complexes in the Rag-Ragulator pathway produced acutely by feeding and how these are altered by alcohol under in vivo conditions. Mice (C57Bl/6; adult males) were fasted, and then provided rodent chow for 30 min (“refed”) or remained food-deprived (“fasted”). Mice subsequently received ethanol (3 g/kg ethanol) or saline intraperitoneally, and hindlimb muscles were collected 1 h thereafter for analysis. Refeeding-induced increases in myofibrillar and sarcoplasmic protein synthesis, and mTOR and S6K1 phosphorylation, were prevented by alcohol. This inhibition was not associated with a differential rise in the intracellular leucine concentration or plasma leucine or insulin levels. Alcohol increased the amount of the Sestrin1•GATOR2 complex in the fasted state and prevented the refeeding-induced decrease in Sestrin1•GATOR2 seen in control mice. Alcohol antagonized the increase in the RagA/C•Raptor complex formation seen in the refed state. Alcohol antagonized the increase in Raptor with immunoprecipitated LAMPTOR1 (part of the Ragulator complex) after refeeding and decreased the association of RagC with LAMPTOR1. Finally, alcohol increased the association of the V_1_ domain of v-ATPase with LAMPTOR1 and prevented the refeeding-induced decrease in v-ATPase V1 with LAMPTOR1. Overall, these data demonstrate that acute alcohol intake disrupts multiple protein–protein complexes within the Rag-Ragulator complex, which are associated with and consistent with the concomitant decline in nutrient-stimulated muscle protein synthesis under in vivo conditions.

## 1. Introduction

Acute alcohol ingestion impairs the global protein synthetic rate in skeletal muscle and can lead to muscle wasting and contractile dysfunction when alcohol use is excessive and sustained (as reviewed in [1,2]). Muscle protein synthesis typically cycles during the course of the day in response to the prevailing nutritional state, decreasing with fasting and increasing after feeding. Alcohol can adversely impact muscle protein synthesis during both of these nutritional phases. In the fasted state, alcohol acutely inhibits TORC1 (mechanistic or mammalian target of rapamycin complex 1) signal transduction, thereby reducing the phosphorylation of S6 kinase (S6K)-1 and eukaryotic initiation factor 4E binding protein (4E-BP)-1 and impairing mRNA translation [3,4]. Alcohol also completely prevents or blunts the mTORC1-mediated enhancement of muscle protein synthesis produced by the branched-chain amino acid leucine [5] in addition to hormones such as insulin and insulin-like growth factor (IGF)-I [4,6]. Although conclusions from these early studies are restricted by the pharmacological nature of the intervention, more recent work reported that acute alcohol ingestion also antagonizes the normal anabolic response to complete nutrient stimulation seen after meal refeeding [7,8].

The physiological interaction of nutrients and hormones on muscle protein synthesis is integrated by the activation of distinct signal transduction pathways [9]. While there is considerable information on the ability of insulin and IGF-I to activate the AKT-TSC2 (tuberous sclerosis complex)-Rheb (Ras homolog enriched in brain) pathway [10], there is a paucity of data on how alcohol impacts the Ras-related GTP-binding (Rag) proteins and other elements of the cellular machinery that senses amino acids. Heterodimers, containing RagA or RagB complexed with RagC or RagD, specifically bind to mTORC1 as opposed to mTORC2 [11]. It is noteworthy that maximal activation of mTORC1 is mediated by a complex of the GTP-bound form of RagA or RagB and the GDP-bound form of RagC or RagD. The GTP loading status of RagA/B in turn is governed by the GTPase-stimulating activity of GATOR1 (GAP activity toward Rag 1) that can repress mTORC1 activity by promoting GTP hydrolysis by RagA and RagB [12]. Adding another layer of complexity, a separate multiprotein complex termed GATOR2 can activate mTORC1 and is regulated by the Sestrin family of proteins, thereby opposing the function of GATOR1 [13]. Finally, the Ragulator is also an essential element of this signaling pathway. It interacts with the Rag proteins redirecting mTORC1 to the surface of the lysosome from the cytoplasm and is thereby selectively activated by amino acids [14]. Although the association of both RagA and RagC with mTOR is decreased in myocytes cultured short-term with alcohol [15], in general there is a lack of information on the effect of alcohol on the Rag-Ragulator complex as a possible mechanism underlying alcohol’s ability to gradually produce the erosion of lean body mass. Moreover, presently most of our understanding of nutrient regulation of mTORC1 is derived from in vitro studies, often using transformed cells with the overexpression or complete knockout of various regulatory proteins. To address this knowledge gap, our study examined the acute effect of alcohol on the abundance of various regulatory protein–protein signaling complexes within the Rag-Ragulator pathway in response to fasting and nutrient stimulation under in vivo conditions.

## 2. Materials and Methods

### 2.1. Animal Protocols

Mice, 12 week old adult males of the C57BL/6 background (Charles River Breeding Laboratories; Cambridge, MA, USA), were housed in-house for 1 week prior to each study. Mice were caged separately under controlled conditions (22 ± 1 °C; 12 h cycle of light/dark; 30–70% humidity) in plastic cages with corncob bedding. Mice were provided ad libitum water and chow (24.3% percent of calories were from protein, 4.7% of calories from fat and 40.2% of calories from carbohydrates (diet 8604 Envigo Teklad, Boston, MA, USA)). Food was removed at approximately 10 p.m., but water was still available. The next morning, at approximately 7 a.m., mice were assigned randomly to the “refed” group, which, for 30 min, was provided unlimited access to chow, or assigned to the “fasted” group, for whom no chow was provided. At the conclusion of the refeeding period, food for the refed group was weighed, and the amount of chow consumed was determined. Next, mice in both groups were randomly divided and either had alcohol injected intraperitoneally (3 g ethanol/kg) or, in the “control” group, were injected with saline (0.9% sterile saline); mice were euthanized 1 h thereafter. Model details have been previously reported [8,16]; in general, food consumption of the control and alcohol-treated mice in the refed groups, likewise, the blood alcohol concentration (BAC) of mice in the fasted alcohol group and the refed alcohol group, did not differ. The amount of alcohol administered was selected because it yields a BAC, at the time of euthanasia, that is similar to what is detected in humans during alcohol intoxication [1].

All experimental procedures adhered to National Institutes of Health (NIH) guidelines for experimental animals and were approved by the Institutional Animal Care and Use Committee of Penn State College of Medicine (PRAMS200746587).

### 2.2. Protein Synthesis in Myofibrillar and Sarcoplasmic Pools

The nonisotopic SUnSET methods were used to semiquantitate in vivo protein synthesis, as originally described [17] and reported by our laboratory [8,16,18]. Mice were injected intraperitoneally (IP) with puromycin (0.04 µmol/g body weight) 30 min prior to euthanasia. The injected puromycin is integrated into elongating peptide chains, and the abundance of newly formed puromycin-labeled peptides is used as a measure of protein synthesis. Next, isoflurane was used to anesthetize mice, and the plantaris and gastrocnemius was removed from each leg and weighed. One muscle was placed in an ice-cold buffer and homogenized, whereas the contralateral muscle was freeze-clamped. All samples were stored at −80 °C. Sarcoplasmic and myofibrillar fractions were then isolated from skeletal muscle [19].

### 2.3. Western Blot and Immunoprecipitation

Homogenates of tissue were mixed with 2× Laemmli SDS buffer after clarification by centrifugation. To assess the relative rates of protein synthesis, samples were electrophoresed on SDS-PAGE and Western blotting was performed. An antibody towards puromycin was used for the detection of peptides containing newly incorporated puromycin and having molecular weights between 20–100 KDa. Specifics related to gel percentage, antibody dilution and the supplier for antibodies used for all Western blots are presented in Supplemental Table S1. In general, proteins were transferred onto PVDF membranes that were incubated at 4 °C overnight with a primary antibody. Blots were developed (Amersham ECL; GE Healthcare Bio-Sciences, RPN2106, Pittsburgh, PA, USA), and then exposed to X-ray film and an intensifying-screen-equipped cassette (DuPont Lightening Plus). NIH Image (version 1.6) was used for scanning and data analysis. Samples from each of the four groups were loaded on the same gel and the data was normalized as a percentage of the value from the fasted control group. However, a direct comparison of relative rates of myofibrillar versus sarcoplasmic protein synthesis is not possible as the two pools were run on separate gels.

So as to maintain protein–protein interactions, tissues were homogenized in a CHAPS (3[(3-cholamidopropyl)dimethylammonio]-propanesulfonic acid) buffer, which included (in mmol/L) 40 HEPES (pH 7.5), 120 NaCl, 1 EDTA, 10 pyrophosphate, 10 β-glycerol phosphate, 50 sodium fluoride, 1.5 sodium vanadate, 0.3% CHAPS and 1 protease inhibitor cocktail. The homogenate was mixed and centrifuged to clarify. A portion of the resulting supernatant was combined with antibodies directed at either raptor (e.g., mTORC1 complex), Mios (e.g., GATOR2 complex) or LAMPTOR1 (e.g., Ragulator complex), and goat antirabbit BioMag IgG beads (PerSeptive Diagnostics, Cambridge, MA, USA) were used to isolate immune complexes. After collection, a CHAPS buffer was used to wash the beads, and SDS-PAGE was performed and analyzed as described above (Table S1).

### 2.4. Leucine, Glutamine, Insulin and Alcohol Concentrations

Precolumn derivatization and reverse phase high-pressure liquid chromatography were used to quantitate the concentration of leucine and glutamine in muscle and plasma [19]. Plasma insulin was assayed using an enzyme-linked immunosorbent assay (Alpco; Salem, New Hampshire; catalog #80-INSMSU-E01), while the blood alcohol level was quantitated with an Analox Instruments rapid analyzer (GL5; Lunenburg, MA, USA).

### 2.5. RNA Extraction and Real-Time Quantitative PCR

In accordance with the manufacturers’ protocols, Tri-reagent (Molecular Research Center, Inc., Cincinnati, OH, USA) and RNeasy mini kits (Qiagen, Valencia, CA, USA) were used for the extraction of total RNA. Tri-reagent was used to homogenize muscle and the subsequent extraction was performed in phenol/chloroform. Seventy percent ethanol was added to the aqueous phase and the sample was then applied to a Qiagen minispin column, and the protocol from the manufacturer was followed [20]. RNA was eluted from the column using RNase-free water and then quantified (Thermo Fisher Scientific, Waltham, MA, USA). RNA quality was assessed using 1% agarose gel, and total RNA was reverse-transcribed (Invitrogen, Carlsbad, CA, USA) as described by the manufacturer. An aliquot of the reverse-transcribed reaction mix was used for qPCR and TaqMan gene expression assays: CAT (cationic amino acid transporter)-1 (NM_013111.2), SNAT (sodium-coupled neutral amino acid transporter)-2 (NM_181090.2), LAT (large neutral amino acid transporter)-2 (NM_019283.1), PAT (proton-coupled amino acid transport)-1 (NM_130415.1), PAT-2 (NM_139339.1), and PAT4 (NM_001108127.1) (Applied Biosystems, Foster City, CA, USA). The endogenous control glyceraldehyde 3-phosphate dehydrogenase (GAPDH, NM_017008.3) was used in the expression of target genes by the 2^−ΔΔCt^ method.

### 2.6. Statistics

Data are presented as the mean ± SEM with individual values presented when appropriate. The sample size for each specific endpoint assessed for each group is provided in the legend for the table or figure. Data were analyzed using a two-way analysis of variance (ANOVA), followed by a post hoc Student–Newman–Keuls test (SNK; Prism 8 for Windows, San Diego, CA, USA). Statistical significance was set at *p* < 0.05.

## 3. Results

**Figure 1:**
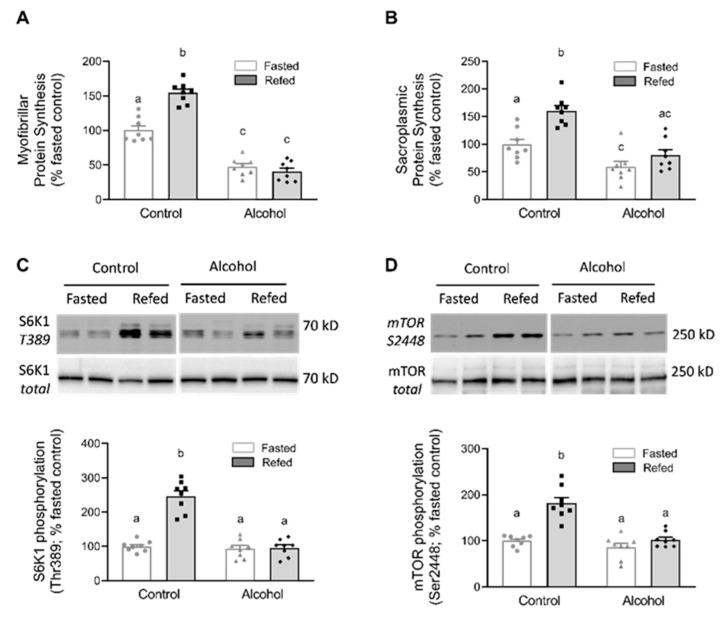
Relative rates of myofibrillar (**A**) and sarcoplasmic (**B**) protein synthesis in fractions isolated from the skeletal muscle of control and alcohol-receiving mice that have been either fasted or refed. Alterations in the total and phosphorylation of S6K-1 and mTOR protein abundance produced by feeding and/or alcohol are also presented (**C**,**D**), respectively. A representative immunoblot for each protein is displayed on top of the respective bar graph summarizing data from all samples analyzed. Although samples from all groups were loaded on the same gel, a gel break depicts lane(s) of the gel that have been deleted. Bar graphs represent analyses for all immunoblots, where the mean of the fasted saline-treated control group was arbitrarily set at 100 arbitrary units (AU). Values are mean ± SEM with *n* = 8/group. Only values having different superscript letters are statistically (*p* < 0.05) different.

The relative rates of muscle protein synthesis were assessed in both the myofibrillar and sarcoplasmic pools as the synthetic rate in these two fractions can be differentially altered. In control animals, there was a coordinate increase in protein synthesis in both protein pools (Figure 1A,B). Acute alcohol also coordinately decreased myofibrillar and sarcoplasmic protein synthesis in the fasted state. After refeeding, sarcoplasmic protein synthesis in alcohol-treated mice was increased to intermediate levels compared to those seen in the fasted control and alcohol-treated rats. In contrast, refeeding did not yield a detectable increase in the myofibrillar pool in alcohol-treated rats. Compared to refed control mice, alcohol blunted the feeding-induced increase in myofibrillar and sarcoplasmic protein synthesis, which was associated with a comparable inhibition of T389 phosphorylation of S6K1, which is an authentic downstream substrate for mTORC1, as well as the inhibition of the S6K1-mediated S2448 phosphorylation of mTOR (Figure 1C,D, respectively).

**Figure 2:**
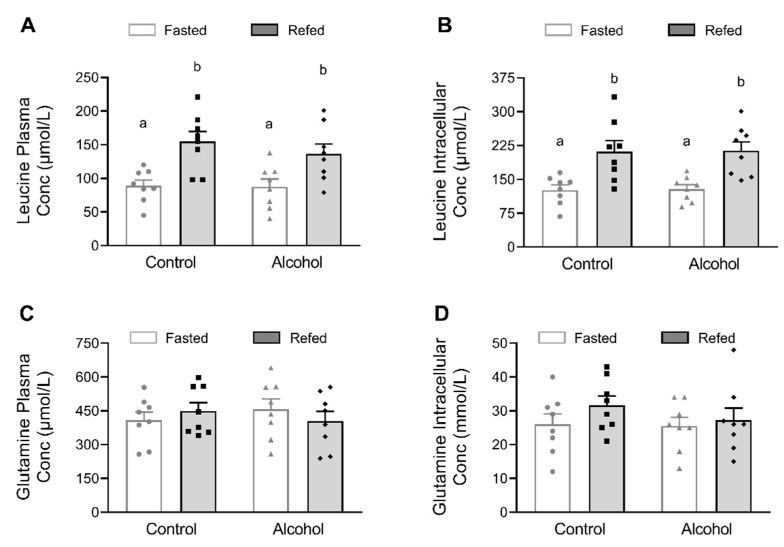
Plasma and intracellular muscle leucine (**A**,**B**) and glutamine concentrations (**C**,**D**) of mice in the control and alcohol-receiving groups under basal or refed conditions. Data are mean ± SEM; *n* = 8/ group. Values having a different superscript letter are statistically (*p* < 0.05) different.

The reduction in mTORC1 activity in alcohol-treated mice after refeeding could result from lower circulating and/or intracellular concentrations of leucine. However, alcohol did not alter the plasma leucine in the basal fasted condition or the increased plasma leucine detected after refeeding (Figure 2A). Likewise, there was no difference in the intracellular leucine content in muscle from control and alcohol-treated mice under either fasting or refed conditions (Figure 2B). Differences between the two alcohol-treated groups could not be explained by differences in BAC as these did not differ between the fasted + alcohol (28 ± 4 mM) and the refed + alcohol (30 ± 5 mM) groups. An increase in plasma or tissue glutamine can also increase protein synthesis [21]. However, there was no effect of alcohol and/or refeeding on either the plasma or intracellular glutamine concentrations in skeletal muscle (Figure 2C,D).

Because the intake of a mixed meal potently stimulates insulin secretion, alcohol might inhibit mTOR1 activity after refeeding by suppressing the insulin response [22]. However, the plasma insulin concentration achieved after refeeding was not different in mice from the control and alcohol-receiving groups (0.83 ± 0.14 ng/mL vs. 0.97 ± 0.19 ng/mL; *p* > 0.05), although insulin levels in both refed groups were higher than their respective fasted control groups (0.31 ± 0.08 ng/mL versus 0.44 ± 0.11 ng/mL).

The intracellular levels of amino acids can be modulated by alterations in cellular transporters for these nutrients, which in turn controls their availability to activate mTORC1 [23]. Table 1 presents data on the effect of alcohol on the abundance of select amino acid transporter mRNAs in muscle from mice in the fasted and refed conditions. At the time point examined, there was no effect of alcohol and/or refeeding on LAT1 and LAT2, which transport branched-chain amino acids; SNAT-2, which enhances cellular uptake of glutamine; CAT1; PAT-1; PAT-2 or PAT-4. As no group differences in mRNA content for these various transporters were detected, subsequent analysis of their protein content was not deemed justified.

**Figure 3:**
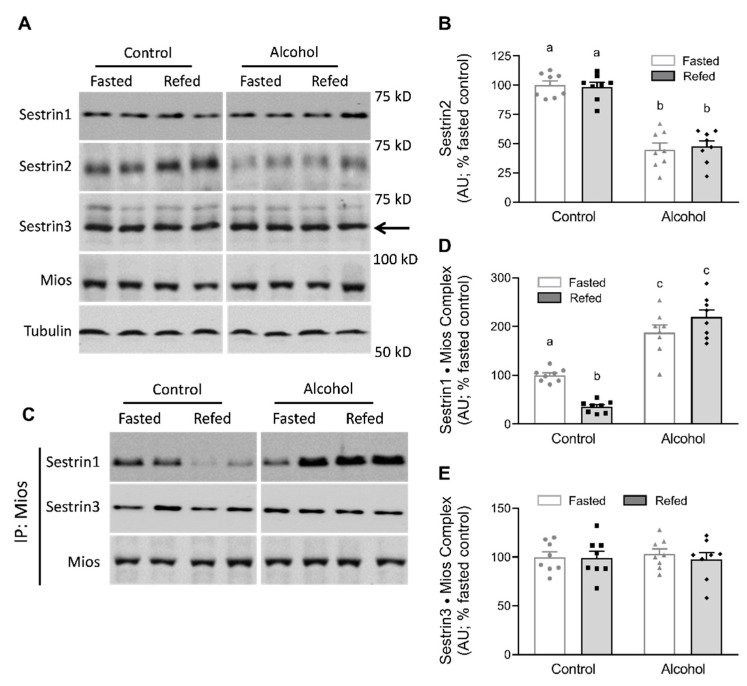
Alterations in Sestrins and GATOR2 after refeeding in muscle from mice in the control and alcohol-receiving groups. *(***A**), representative immunoblots and the associated bar graph summarize data from all samples analyzed for Sestrin2 (**B**). For Sestrin1, Sestrin3 and Mios, there were no treatment effects (*p* > 0.05), and mean data are not shown. Although samples from all groups were loaded on the same gel, a gel break depicts lane(s) of the gel that have been deleted. In (**C**), Mios (protein component of the GATOR2 complex) was immunoprecipitated (IP) and Western blotting performed for Sestrin-1, -2, -3 and Mios. Under our assay conditions, we could not detect binding of Sestrin2 to Mios (data not shown); therefore only the association of Sestrin-1 and -3 to the GATOR2 subunit Mios was quantitated (**D**,**E**), respectively). Bar graphs represent the analyses for all immunoblots, where the mean of the fasted saline-treated control group was arbitrarily set at 100 AU. Values are mean ± SEM with *n* = 8/group. Only values having different superscript letters are statistically (*p* < 0.05) different.

Distinct lines of evidence demonstrate that the Sestrin family of proteins are important elements in the pathway, sensing amino acids and regulating mTORC1 activity by modulating Rag guanine nucleotide exchange [11,24]. Western blot analysis indicated no significant treatment effect on the relative amount of Sestrin1, Sestrin3 and Mios (a protein subunit of GATOR2 complex) in muscle (Figure 3A). However, acute alcohol reduced Sestrin2 protein content by ≈50%, independent of nutritional state (Figure 3A,B). Sestrins can also interact in an amino acid-dependent manner with the GATOR2 complex to inhibit its activity [24]. Therefore, we evaluated the association of GATOR2 with Sestrin by immunoprecipitating Mios, which, in conjunction with WDR24 and WDR59, constitutes the GATOR2 complex. As illustrated in Figure 3C,E, there was a reduction in the amount of Sestrin1 coimmunoprecipitated with Mios after refeeding in muscle from control mice. In contrast, the amount of Sestrin1 bound to GATOR2 was greater in fasted mice administered alcohol, compared to mice in the fasted control group. Moreover, unlike the refed control mice, no decrease was observed in refed mice that subsequently received alcohol. Additionally, there was no alcohol and/or refeeding-induced change in the association of Sestrin3 with GATOR2. Finally, given the limits of our assay, we were unable to reliably detect the binding of Sestrin2 to GATOR2 in skeletal muscle (data not shown). Lastly, we assessed the relative protein abundance for the various subunits within the GATOR1 complex that are involved in the inhibition of mTORC1 [25]. In this regard, we failed to detect any treatment effect on the abundance of DEPDC5, NPRL2 or NPRL3 protein among the various groups (data not shown).

**Figure 4:**
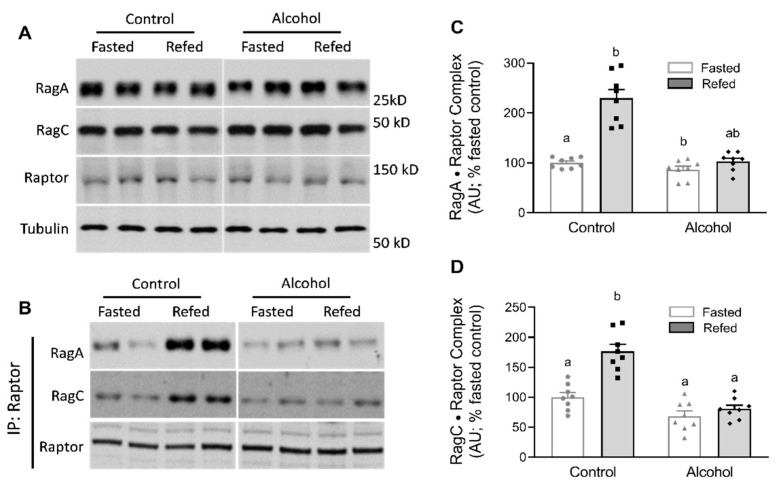
Alterations in Raptor, RagA and RagC after refeeding in the muscles of control and alcohol-receiving mice. (**A**), representative immunoblots for each. For RagA, RagC and Raptor, there were no significant treatment effects (*p* > 0.05), and mean data are not shown. Although samples from all groups were loaded on the same gel, a gel break depicts lane(s) of the gel that have been deleted. In (**B**), Raptor was immunoprecipitated (IP) and Western blotting was performed on RagA (**C**), RagC (**D**) bound to Raptor. Bar graphs represent analyses for all immunoblots, where the mean of the fasted saline-treated control group was arbitrarily set at 100 AU. Values are mean ± SEM with *n* = 8/group. Only values having different superscript letters are statistically (*p* < 0.05) different.

Downstream of Sestrin-GATOR2 binding, the Ras-related small GTP-binding Rag proteins also interact with mTORC1 and stimulate amino acid-dependent protein synthesis by promoting mTORC1 translocation to the lysosome [11]. As there is direct interaction between Raptor in the mTORC1 and the Rag proteins, we next examined the relative abundance of Raptor and the Rag proteins as well as the formation of Raptor•Rag protein complexes. Although mTOR is present in both mTORC1 as well as mTORC2, the defining component for the former multiprotein complex is Raptor. There was no detectable effect of feeding and/or alcohol on the total abundance of Raptor or RagA and RagC (Figure 4A). In contrast, after refeeding, the relative amount of both RagA and RagC bound to Raptor increased in control mice (Figure 4B–D). Compared to values from control mice, a 2-way ANOVA indicated an alcohol effect (*p* < 0.01) for the relative abundance of RagA•Raptor and RagC•Raptor, though only the abundance of the RagA•Raptor complex proved to be statistically significant upon post hoc SNK analysis between the control and alcohol-receiving mice after refeeding. There was no increase in the abundance of the RagA/C•Raptor complex in alcohol-treated mice that were refed when compared to values from control mice.

**Figure 5:**
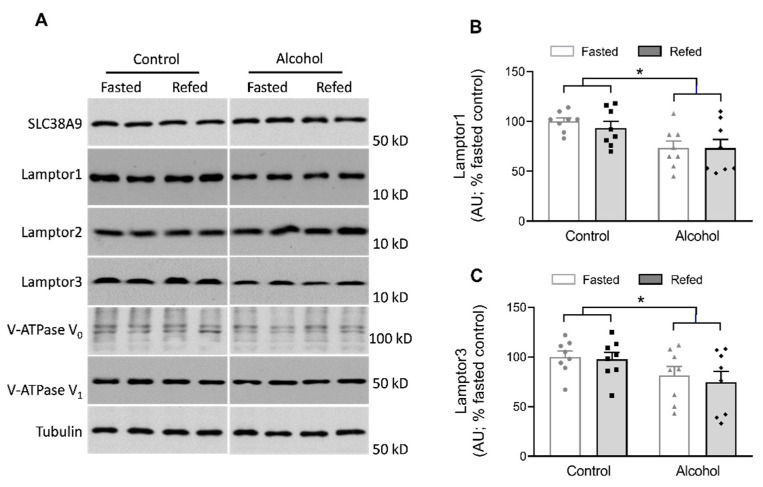
Alterations in Ragulator complex subunits after refeeding in the muscles of control and alcohol-receiving mice. (**A**), representative immunoblots for each. With the exception of LAMPTOR1 and LAMPTOR3, none of the proteins shown demonstrated a significant (*p* > 0.05) alcohol effect using a 2-way ANOVA; hence, mean data are not shown. Although samples from all groups were loaded on the same gel, a gel break depicts lane(s) of the gel that have been deleted. (**B**,**C**) are bar graphs of all immunoblots for LAMPTOR1 and LAMPTOR3 that showed an alcohol effect on the abundance of these two proteins. Bar graphs represent analyses for all immunoblots, where the mean of the fasted saline-treated control group was arbitrarily set at 100 AU. Values are mean ± SEM with *n* = 8/group. * *p* < 0.05, alcohol treatment effect compared to control values.

Additionally, the multiprotein Ragulator complex (i.e., composed of p18, p14, MP1, C7orf59 and HBXIP, otherwise known as LAMPTOR (late endosomal/lysosomal adaptor MAPK and mTOR activator) 1 through 5) anchors Rag heterodimers to the lysosome, which is compulsory for the activation of mTOR by amino acids [26]. There were no alterations in total abundance of LAMPTOR1–3 or SLC38A9 produced by feeding in either control or alcohol-receiving mice (Figure 5A).

**Figure 6:**
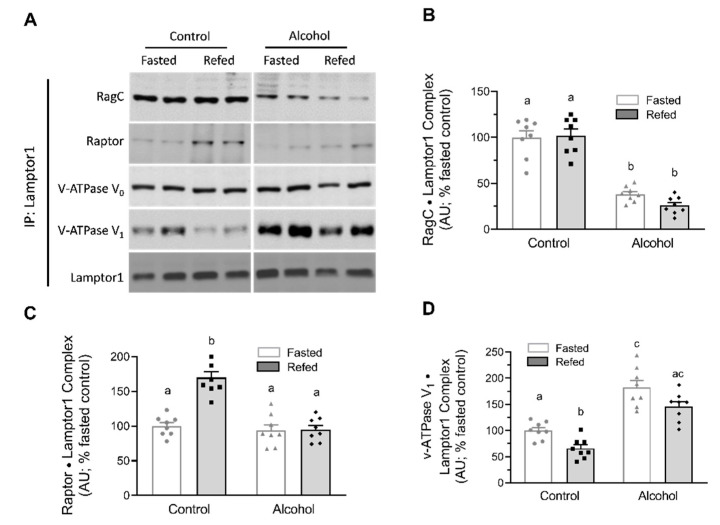
Alterations in LAMPTOR1 protein complexes after refeeding in muscles from control and alcohol-receiving mice. LAMPTOR1 was immunoprecipitated (IP) and immunoblotting was performed for RagC, Raptor and v-ATPase V_1_. (**A**) presents representative Western blots for these complexes. Although samples from all groups were loaded on the same gel, a gel break depicts lane(s) of the gel that have been deleted. Data from all tissues are quantitated in bar graphs (**B**–**D**). Bar graphs represent analyses for all immunoblots, where the mean of the fasted saline-treated control group was arbitrarily set at 100 AU. Values are mean ± SEM with *n* = 8/group. Only values having different superscript letters are statistically (*p* < 0.05) different.

In contrast, while a 2-way ANOVA indicated that there was a significant alcohol effect on both LAMPTOR1 and LAMPTOR3 protein content in muscle (*p* < 0.05; Figure 5B,C), there were also no changes in the relative abundance of the peripheral (V_1_) and integral membrane-associated (V_0_) domains of the vacuolar proton V-ATPase (v-ATPase), which functions as a proton pump in the muscle homogenate from alcohol-treated and/or refed mice (Figure 5A). When LAMPTOR1 was immunoprecipitated, refeeding of control mice increased the amount of Raptor bound to LAMPTOR1 and decreased the amount of RagC and v-ATPase V_0_ bound to LAMPTOR1 (Figure 6A–D). In the fasted state, alcohol increased the binding of RagC and v-ATPase V_0_ to LAMPTOR1. Finally, alcohol prevented alterations in the association of Raptor, RagC and v-ATPase V_0_ to LAMPTOR1 after feeding that were seen in refed control mice.

## 4. Discussion

Although previous research demonstrates that alcohol can antagonize the ability of amino acids and hormonal growth factors to upregulate muscle protein synthesis by inhibiting the multicomponent kinase mTORC1 [1,2], the exact mechanism for this anabolic resistance remains elusive. Our data reveal alcohol acutely alters various protein–protein interactions within the Rag-Ragulator complex which are consistent with alcohol’s inhibitory action on mTORC1 (e.g., decreased S6K1 phosphorylation) and global protein synthesis after nutrient stimulation. The physiological importance of this increased S6K1 has been previously confirmed by data showing the increased phosphorylation of authentic substrates S6, eukaryotic elongation factor (eEF)-2 kinase and eukaryotic initiation factor (eIF)-4B, which would increase mRNA translation initiation and elongation [8]. Our data also reveal refeeding increases the global synthesis of proteins in the myofibrillar and sarcoplasm pools in muscle, and that alcohol acutely inhibits the synthetic response in both protein pools.

The refeeding paradigm used in the current in vivo study transiently increases both insulin and leucine, which can function separately or together to enhance protein synthesis [27]. However, the prevailing plasma concentrations of insulin and leucine were not different between the control mice and those that received alcohol and were refed. Such data imply alcohol’s effect is mediated directly on muscle and is consistent with data generated from cultured myocytes and the in vitro epitrochlearis muscle preparation indicating the ability of ethanol to directly antagonize hormone- or amino acid-stimulated protein synthesis and S6K1 activity in muscle [15,28]. Moreover, alcohol did not acutely change the increased intracellular content of leucine in refed mice. Changes in intracellular amino acids can alter the expression and/or activity of various amino acid transporters and produce concomitant changes in mTORC1 activity and muscle growth [29,30,31]. However, in the current study, we were unable to detect alcohol- and/or nutrient-induced changes in the mRNA expression of LAT1 and SNAT2, as well as of other amino acid transporters such as LAT2, CAT1 or PAT1-, -2 or -4. Furthermore, muscle contains a functional bidirectional transport system wherein SNAT2 and LAT1 function in combination to export glutamine while increasing the influx of leucine [32], and changes in intracellular glutamine can impact mTORC1 activity [33]. However, again, we did not detect a significant alteration in the intracellular glutamine concentration after acute alcohol or refeeding. Our results differ from those reported in healthy human subjects where the infusion of essential amino acids increased the mRNA expression of PAT1, SNAT2 or LAT1 within 1 h [33]. This apparent discrepancy may have resulted from the significantly greater (nearly 10-fold) increase in plasma leucine in the later study, compared to the more physiological 2-fold rise seen in the current study. Collectively, our results suggest that the alcohol-induced decrease in mTORC1 activity, in both the basal and refed condition, are not due to limited intracellular amino acid availability. As a result of these data, we examined key protein–protein interactions within the Rag-Ragulator signaling pathway as a possible mechanism for this inhibitory action of alcohol on muscle protein synthesis.

The Rag GTPases are Raptor (mTORC1)-interacting proteins important for the activation of mTORC1 by amino acids, independent of the insulin-stimulated AKT-TSC1/2-Rheb pathway, and are essential for the translocation of mTORC1 to the lysosome [11]. Our data in muscle from control mice indicate refeeding increases the amount of RagA and RagC bound to Raptor. This response is comparable to the increased Rag•Raptor complex formation detected after a 2 h infusion of insulin or a balanced mixture of amino acids [34], after 24 h of a high-protein diet or a low-protein diet that contained leucine supplementation [35], and in myocytes cultured with increased leucine [15]. Importantly, alcohol largely prevented the increased formation of the RagA•Raptor and RagC•Raptor complexes in refed mice. This inability to form functional Rag•Raptor complexes is consistent with the nutrient-resistant condition produced by other catabolic insults associated with impaired mTORC1 activity and decreases in muscle protein synthesis [20,35]. Likewise, alcohol decreased the binding of RagC with LAMPTOR1, one of the proteins within the pentameric Ragulator complex. This Ragulator complex is essential for amino acid-specific activation of mTORC1, and in its absence, the Rag proteins fail to target mTORC1 to the lysosome [14,26]. However, our results differ from those obtained using cell-based systems wherein amino acid or leucine deprivation (e.g., metabolic stress) strengthened the association between RagC and LAMPTOR1 [14]. The reason for these apparently discordant results is uncertain, and there are no previous reports in which this protein–protein interaction has been examined under in vivo conditions where proteins have been either knocked out or overexpressed.

The proton v-ATPase is a multisubunit complex that, in conjunction with the Rag GTPases and the Ragulator proteins, forms a physical supercomplex essential for amino acid-generated stimulation of mTORC1 [36]. The peripheral V_1_ domain of v-ATPase carries out ATP hydrolysis, with the V_0_ domain regulating proton translocation and the acidification of the lysosomal lumen [37]. Our results demonstrate an increase in the binding of v-ATPase V_1_ with LAMPTOR1 in response to alcohol, both under basal fasted conditions as well as after feeding. Moreover, there was no change in the association of LAMPTOR1 with V_0_ domain of v-ATPase in response to nutrients or alcohol. These results are consistent with in vitro observations wherein amino acids decreased the abundance of LAMPTOR1 (p18) bound to v-ATPase V_1_, but not the V_0_ domain [14].

The Sestrin family of protein is recognized to be authentic intracellular leucine sensors, and the sequestration of intracellular leucine by Sestrin isoforms can disrupt the Sestrin•GATOR2 complex, thereby activating mTORC1 [12,38]. Others have reported a decrease in Sestrin2•GATOR2 complex abundance after dietary supplementation with leucine or high-protein [35] or short-term infusion of amino acids [34]. In our current study, while we detected an alcohol-induced decrease in total Sestrin2, we could not detect binding of Sestrin2 to one of the proteins in the GATOR2 complex (e.g., Mios). However, refeeding did lead to the dissociation of endogenous Sestrin1 from GATOR2 in muscle from control mice, consistent with the leucine-induced decrease in the Sestrin1•GATOR2 complex seen under in vitro conditions [38]. Moreover, alcohol acutely increased the relative abundance of Sestrin1•GATOR2 (e.g., decreased dissociation of Sestrin1 from GATOR2) in both the fasted and refed state. As Xu et al. [38] has previously reported, Sestrin1 abundance is substantially greater than Sestrin2 in muscle, and there was no nutrient-induced change in the abundance of Sestrin3•GATOR2 complex (present study and [38]). Our data are consistent with refeeding producing a leucine-induced dissociation of Sestrin1 from GATOR2 and the concomitant decrease in mTORC1 activity and muscle protein synthesis seen in alcohol-treated mice.

This paper provides a comprehensive account of alcohol’s effect on protein–protein interactions regulating skeletal muscle protein synthesis via the Rag-Ragulator signal transduction pathway in response to a physiologically relevant nutritional mixed-meal stimulus. These data extend knowledge of this signaling network under in vivo conditions during the normal fasting-to-fed nutrient transition, as well as during the pathophysiological stress condition of acute alcohol intoxication. Alcohol’s ability to suppress the typically observed increase in muscle mTORC1 activity in response to food was temporally associated with the disruption of multiple protein–protein interactions within the Raptor-Rag-Ragulator supercomplex. While we cannot ascribe causality, our data are largely consistent with mechanistic studies performed in various cell lines in response to amino acid-sufficient and -deficient conditions. Hence, while it remains possible that a portion of alcohol’s ability to decrease protein synthesis and mTORC1 activity occurs by other mechanisms that are independent of the Rag GTPase-Regulator pathway [39], our data are consistent with defects in this pathway negatively impacting synthetic rates of proteins in both myofibrillar and sarcoplasmic pools in the basal state and acutely after food intake. While the conclusion from this study are limited to its short time frame, we speculate that, if such alcohol-induced changes are sustained over relatively long periods of time, they may be at least partially responsible for the gradual development of skeletal muscle myopathy with sustained alcohol consumption.

## Figures and Tables

**Table 1:** Effect of alcohol and refeeding on amino acid transporter mRNA content in muscle.

	Control		Alcohol	
	Fasted	Refed	Fasted	Refed
LAT1 (slc7a5)	1.00 ± 0.11	1.25 ± 0.11	0.92 ± 0.05	1.11 ± 0.24
LAT2 (slca2)	1.00 ± 0.07	0.99 ± 0.09	1.11 ± 0.18	0.94 ± 0.14
SNAT2 (slc38a2)	1.00 ± 0.05	1.04 ± 0.15	1.06 ± 0.12	1.03 ± 0.08
CAT1 (slc7a1)	1.00 ± 0.11	0.89 ± 0.24	0.98 ± 0.12	1.05 ± 0.12
PAT-1 (slc36a1)	1.00 ± 0.07	0.93 ± 0.09	0.97 ± 0.08	0.92 ± 0.09
PAT-2 (slc36a2)	1.00 ± 0.12	1.08 ± 0.12	1.13 ± 0.21	1.08 ± 0.11
PAT-4 (slc36a4)	1.00 ± 0.08	0.97 ± 0.10	0.94 ± 0.11	1.04 ± 0.08

Data are means ± SEM; *n* = 8/group. The relative mRNA content for each transporter has been normalized to GAPDH and then as a percentage of the fasted control group. A two-way ANOVA indicated no treatment effect for mRNA abundance for any of the amino acid transporters assessed.

## Supplementary Materials

The following are available online at https://www.mdpi.com/article/10.3390/nu13041236/s1, Table S1: Antibodies and gel details for Western blot analysis.
