# Supplementary material for: Alcohol Acutely Antagonizes Refeeding-Induced Alterations in the Rag GTPase-Ragulator Complex in Skeletal Muscle
Source: Nutrients. 2021 Apr 9;13(4):1236. doi: 10.3390/nu13041236 (PMC8070399; doi:10.3390/nu13041236)
Supplement: Supplementary file 1 [file nutrients-13-01236-s001.pdf]

Supplement Table S1. *Antibodies and gel details for Western blot analysis*

Antibody	Vendor	Catalog no.	Dilution	Expected MW (KDa)	SDS-PAGE gel (%)	Protein loaded (µg)
**Actin**	CST	8457	1:10,000	45	10	50
**DEPDC5**	Abcam	Ab185565	1:4000	170-180	4-20% criterion	20
**Folliculin**	CST	3697	1:1000	70	6	90
**GAPDH**	CST	2118	1:10,000	37	12.5	25
**GATOR1 (NPRL2)**	CST	37344	1:1000	41	12.5	50
**GATOR2 (Mios)**	CST	13557	1:1000	98	10	50
**GATSL3**	SC	377385	1:1000	36	15	50
**LAMTOR1**	CST	8975	1:1000	18	15	50
**LAMTOR2**	CST	8145	1:1000	14	15	90
**LAMTOR3**	CST	8168	1:1000	14	15	90
**LAT1**	CST	5347	1:1000	39	15	50
**mTOR total**	CST	2972	1:1000	289	6	90
**mTOR (S2448)**	CST	2971	1:1000	289	6	90
**NDRG1 (T346)**	CST	5482	1:2000	46,48	10	50
**Puromycin**	Millipore	MABE343	1:4000	15-250	15	20
**RagA**	CST	4357	1:1000	30	12.5	50
**RagC**	CST	9480	1:1000	50	10	50
**Raptor**	CST	2280	1:2000	150	6	90
**Rheb**	CST	13879	1:1000	16	15	90
**Sestrin -1**	Proteintech	21668-1-AP	1:1000	66-68	15	50
**Sestrin-2**	Proteintech	10795-1-AP	1:1000	54-60	7.5	50
**Sestrin-3**	Abgent	AP12471c	1:1000	57	10	50
**SGK1 (S422)**	Abcam	55281	1:1000	49	10	50
**S6K1 (T389)**	CST	9234	1:1000	70,85	7.5	50
**S6K1 total**	SC	230	1:10,000	70,85	12.5	37
**SLC38A9**	Ther-moFisher	PA5-69930	1:1000	64	10	50
**Tubulin**	CST	2128	1:10,000	55	7.5	90
**v-ATPase – VOA2**	Sigma-Al-drich	SAB2701067	1:1000	98	7.5	50
**V-ATPase – VB1/2**	SC	271832	1:500	56	7.5	50

Abcam (Cambridge, UK); Santa Cruz Biotechnology (Dallas, TX); CST, Cell Signaling Technology (Danvers, MA), DSHB, Developmental Studies Hybridoma Bank (Iowa City, Iowa); Invitrogen/ThermoFisher (Carlsbad, CA); Sigma (St. Louis, MO). MW, molecular weight.
